# Direct detection of photoinduced magnetic force at the nanoscale reveals magnetic nearfield of structured light

**DOI:** 10.1126/sciadv.add0233

**Published:** 2022-11-09

**Authors:** Jinwei Zeng, Mohammad Albooyeh, Mohsen Rajaei, Abid Anjum Sifat, Eric O. Potma, H. Kumar Wickramasinghe, Filippo Capolino

**Affiliations:** ^1^ Wuhan National Laboratory for Optoelectronics and School of Optical and Electronic Information, Huazhong University of Science and Technology, Wuhan, Hubei 430074, China.; ^2^ Department of Electrical Engineering and Computer Science, University of California, Irvine, Irvine, CA 92697, USA.; ^3^ Mobix Labs Inc., 15420 Laguna Canyon, Irvine, CA 92618, USA.; ^4^ Department of Chemistry, University of California, Irvine, Irvine, CA 92697, USA.

## Abstract

We demonstrate experimentally the detection of magnetic force at optical frequencies, defined as the dipolar Lorentz force exerted on a photoinduced magnetic dipole excited by the magnetic component of light. Historically, this magnetic force has been considered elusive since, at optical frequencies, magnetic effects are usually overshadowed by the interaction of the electric component of light, making it difficult to recognize the direct magnetic force from the dominant electric forces. To overcome this challenge, we develop a photoinduced magnetic force characterization method that exploits a magnetic nanoprobe under structured light illumination. This approach enables the direct detection of the magnetic force, revealing the magnetic nearfield distribution at the nanoscale, while maximally suppressing its electric counterpart. The proposed method opens up new avenues for nanoscopy based on optical magnetic contrast, offering a research tool for all-optical spin control and optomagnetic manipulation of matter at the nanoscale.

## Teaser

Structured light–induced magnetic force detection leads to an accurate magnetic nearfield characterization.

## INTRODUCTION

Modern optical nanoelements exhibit ever-smaller sizes and individualized functionalities. Those elements that can efficiently manipulate the magnetic field of light boast promising future applications with a great challenge: The magnetic nearfield cannot be directly measured with farfield characterization methods.

Conventional optical devices usually detect and manipulate light in the farfield, i.e., at distances that are optically far from external and/or induced light sources. In the farfield, radiation is devoid of evanescent wave components and solely travels in the form of propagating waves. Consequently, optical sensing in the farfield lacks the subwavelength information that is present in the nearfield of light-matter interaction and is heavily influenced by nonlocal effects (*1*–*3*). In principle, farfield optical devices are diffraction limited and are prone to environmental background noise (*3*). Therefore, measurements performed with farfield optical devices have intrinsically limited resolution and signal-to-noise ratio (SNR). The development of nanotechnology, however, forges new ways of light manipulation, where light-matter interactions are controlled and probed in the nearfield (*3*–*8*). Nearfield optical devices enable local manipulations at the subwavelength scale, free from interference with background radiation (*3*).

Modern nearfield optical nanodevices commonly interact with the electric component of light (*9*), while the possibility to create optical magnetic nano devices provides tantalizing opportunities that are yet to be fully exploited. For example, the excitation and manipulation of the optical magnetic dipole in lanthanides can reach the molecular level (*4*, *10*), so that future magnetic storage devices could be made from single lanthanide nanostructures with all-optical read/write capacity (*11*). In addition, the use of optical magnetic force at the nanoscale in optical magnetic tweezers, magnetic levitations, etc. would offer new means for the accurate mechanical control of nanoscale samples compared to conventional mechanical technologies (*12*, *13*). Therefore, it would be very beneficial to have a method that enables the exploitation of the elusive magnetic response of matter and can even manipulate the usually overshadowed magnetic nearfield (*14*, *15*). As a result, the pursuit of these promising opportunities demands a reliable method for directly characterizing the magnetic component of nearfield light with nanoscale accuracy.

Here, we place a special emphasis on the “direct and exclusive” detection of the optical magnetic field as an essential component for the aforementioned important applications, which, in principle, cannot be simply replaced by optical electric detection. Although the magnetic part of light can be indirectly retrieved via optical electric detection by using Maxwell’s equations, these retrieval procedures require detailed knowledge of the phase, polarization, and magnitude of the electric nearfield. The information needed for retrieval is typically unavailable in optical measurements because of technological constraints, and actual retrieval computations can be very elaborate.

Challenges arise because either the electric and magnetic fields are always coupled in the farfield with a ratio given by the local electromagnetic field impedance, or the magnetic response of common materials is overwhelmed by the electric response at optical frequencies (*16*–*20*). In the nearfield generated by electric dipoles, the wave impedance is much higher than the corresponding situation in free space, making it even more difficult to investigate the magnetic field exclusively. As a result, most detectors developed so far are based on matter interaction only with the electric component of light, which can be considered as an incomplete exploitation of electromagnetic properties.

To address this challenge, we provide experimental evidence of exclusive magnetic force detection at the nanoscale, made possible through the force exerted on a magnetic dipole induced by the magnetic component of light, as theoretically predicted in (*21*, *22*). The detection of the magnetic force at optical frequencies is based on generating a photoinduced magnetic dipole in a magnetically polarizable nanoprobe. The magnetic nanoprobe used here to measure the force is an Si truncated cone, which features low dissipative losses at optical frequencies and benefits from practical fabrication protocols. In addition, we use an azimuthally polarized beam (APB) for illuminating the nanoprobe. This form of structured light features a polarization singularity with a vanishing electric field and a maximum longitudinal magnetic field on axis (*23*).

We show experimentally that the magnetic nanoprobe exhibits a degree of azimuthally geometrical symmetry that guarantees an exclusive optical magnetization and minimizes the electric dipole and quadrupole when excited by an APB. We then show how the resulting magnetization is directly related to the magnetic component of light of the tightly focused APB.

One of the main goals of this paper is the experimental demonstration of the detection of the magnetic gradient force. We also show that the magnetic nanoprobe provides an exclusive detection of the local magnetic field rather than the electric field, a property derived from the mutual symmetry between the nanoprobe structure and the illumination beam. Although the principle of magnetic excitation of a resonating nanostructure was already demonstrated in (*24*), the possibility of using such a nanostructure for magnetic force detection has not yet been experimentally explored. In this work, we design, fabricate, and implement the Si truncated cone as the magnetic nanoprobe in photoinduced force microscopy (PiFM) to detect the magnetic force of light exclusively and directly. With the aid of this probe, we prove that the measured optical force is dominated by the photoinduced magnetic force, which is generated by the incident magnetic field. In summary, here, we demonstrate (i) the capability to measure the magnetic optical force and (ii) that the proposed scanning magnetic nanoprobe system enables direct and exclusive registration of the magnetic nearfield of structured light at the nanoscale.

## RESULTS

### Principle of magnetic force detection

The proposed method registers the photoinduced magnetic force, i.e., the force acting on a photoinduced magnetic dipole in a scanning nanoprobe, induced by the local magnetic component of light. The operating principle is inspired by the photoinduced (electric) force microscope (PiFM), which typically uses a gold-coated tip in a bottom- or side-illuminated atomic force microscope to accurately sense the electric nearfield distribution (*3*, *23*, *25*–*27*). To detect the magnetic component of light, the gold-coated tip used in PiFM can be replaced by a structure that exhibits a high magnetic polarizability at optical frequencies, i.e., a magnetic nanoprobe. Previously, we have designed and demonstrated an Si truncated cone structure that has a Mie-type magnetic resonance (*24*) as an ideal candidate of the magnetic nanoprobe. A Mie-type magnetic resonance is achieved through assembling geometries of highly refractive dielectric nanoscatterers, which display circulating electric displacement currents that are associated with a magnetic dipole moment (*28*, *29*). We choose Si as the tip material because of the low dissipative loss of Si and the ease of fabrication afforded by the use of commercially available Si probe tips as a template.

To enhance the excitation of the magnetic resonance, we use structured light to illuminate the probe. Under APB illumination, the magnetic mode of the Si truncated cone is exclusively excited (*21*, *22*, *24*). Because the APB has an azimuthally symmetric electric field distribution that circulates about its beam axis and fully vanishes at the beam center, it exhibits an axial (longitudinal) magnetic field (*30*, *31*). The magnetic field component of the APB induces the signature circulating displacement current inside the Si truncated cone. When the cone is properly aligned with the beam axis, this geometry provides the exclusive Mie-type magnetic resonance excitation and forms the photoinduced magnetic dipole oriented along the longitudinal direction, i.e., colinear with the beam propagation axis (*24*). In the case when the Si truncated cone and the incident beam are slightly misaligned, we show that the magnetic response of the nanoprobe is still dominant compared to the electric response, underlining the potential robustness of the method to setup tolerances (*24*).

**Fig. 1.**
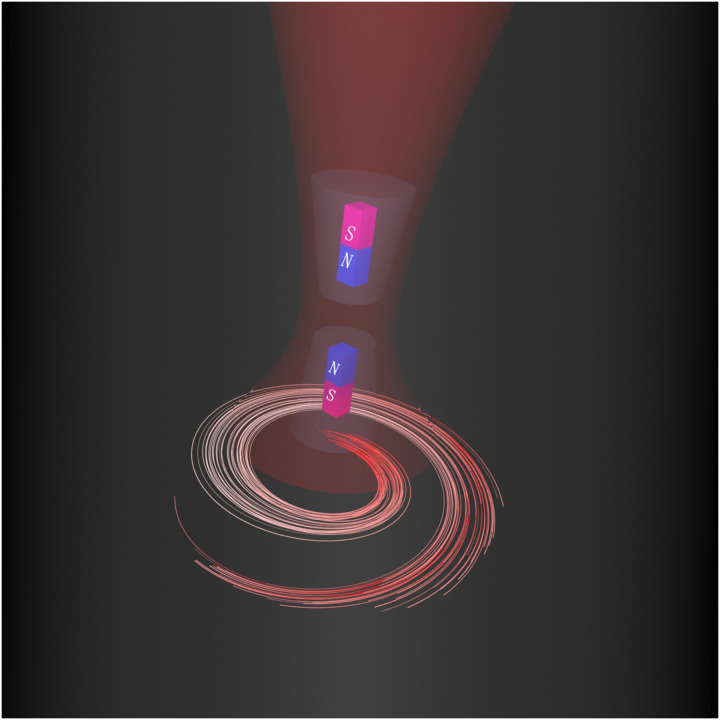
The concept of photoinduced magnetic force detection. Structured light with azimuthal symmetry and dominant axial magnetic field induces magnetic dipoles in two magnetic polarizable nanoparticles, and an exclusive magnetic dipolar force is created between them.

**Fig. 2.**
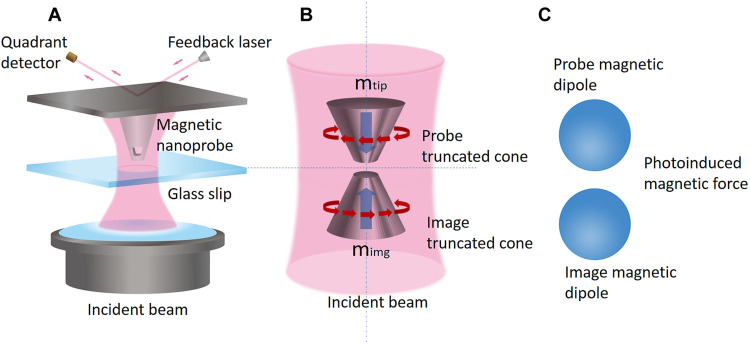
Schematic illustration of photoinduced magnetic force detection. (**A**) Schematic of the PiFM instrument used in this work. (**B**) Zoom-in region of the Si truncated cone working as photoinduced magnetic nanoprobe and its image in the glass slip (or substrate). The rotating arrows represent the excited electric field under the magnetic resonance condition, and the bold blue arrows show the directions of probe and image magnetic dipoles m_tip_ and m_img_, respectively. (**C**) Two photoinduced magnetic polarized nanoparticles exerting a magnetic force on each other.

The concept and schematic of the magnetic force technique are illustrated in Figs. 1 and 2, respectively. The incident APB is tightly focused by the bottom oil-immersion objective lens through a glass coverslip onto the Si truncated cone (i.e., the magnetic nanoprobe) as indicated in Fig. 2A. The incident APB excites the magnetic mode in the Si probe, inducing a magnetic dipole. When the nanoprobe approaches the glass slip (or substrate) within a few nanometers’ distance, the interaction between the nanoprobe and the substrate under APB illumination is modeled with two interacting magnetic dipoles: the induced magnetic dipole in the nanoprobe and its image, which accounts for the substrate effect, as indicated in Fig. 2B. Such a system of two closely spaced magnetic dipoles is shown in Fig. 2C and generates a magnetic dipole-dipole interaction force, which is the observable in this technique.

### From magnetic field to magnetic force

In the photoinduced magnetic force measurement, the incident magnetic field is extracted from the measured total optical force. For this purpose, we analyze the physical origin of the optical force exerted on a nanoprobe in force microscopy. In general, the optical force on an object is due to the Lorentz force density, provided by the distribution of the induced charge and current densities ρ_ind_(**r**, *t*) and ***J***_ind_(**r**, *t*), respectively. The Lorentz force density ***f***(**r**, *t*) exerted at any position **r** of an object, at time *t*, subject to electromagnetic field with electric field ***E***(**r**, *t*) and magnetic induction ***B***(**r**, *t*) reads ***f***(**r**, *t*) = ρ_ind_***E*** + ***J***_ind_ × ***B***. Here, time-dependent quantities are in italic. Therefore, the total force on an object with volume *V* is given by $F_{\text{tot}}^{\text{Lorentz}}(t)=\int_{V}f\;\mathrm{dv}$. Considering time-harmonic fields with e^−*i*ω*t*^ dependence, using phasors (letters with a normal font), the time-average total optical force is given by $F_{\text{tot}}^{\text{Lorentz}}=\frac{1}{2}\text{Re}(\int_{V}\rho_{\text{ind}}E^{*}+J_{\text{ind}}\times B^{*}\mathrm{dv})$.

Next, we assume that the nanoprobe response is well approximated by its electric and magnetic dipolar responses; hence, we neglect the contributions from higher-order multipoles. The proof for the validity of this assumption is given in the Supplementary Materials. Therefore, the time-average total optical force reduces to (*13*, *32*)

$$F_{\text{tot}}^{\text{dipole}}=\frac{1}{2}\text{Re}{\left\{(\nabla E^{\text{loc}}(r)\right)}^{*}\cdot p+\mu_{0}{\left(\nabla H^{\text{loc}}(r)\right)}^{*}\cdot m-\mu_{0}\frac{{\mathrm{ck}}^{4}}{6\pi }(p\;\times \;m^{*})\}$$ (1)

Here, ∇**E**^loc^ and ∇**H**^loc^ are the gradient of the local electric and magnetic fields, respectively; **p** and **m** denote the electric and magnetic dipole moments in the nanoprobe, respectively (see eq. S5); *c* is the speed of light in vacuum, *k* is the free-space wave number, μ_0_ is the free-space permeability; and the superscript * denotes complex conjugation. When the nanoprobe is a photoinduced dominant electric or magnetic dipole (with one dominating on the other), the last term μ_0_ *ck*^4^(**p** × **m**^∗^)/(6π) in Eq. 1 is negligible. This yields $F\approx F_{p}^{\text{dipole}}+F_{m}^{\text{dipole}}$, where the corresponding photoinduced electric and magnetic dipolar force expressions are

$$F_{p}^{\text{dipole}}=\frac{1}{2}\text{Re}\{(\nabla E^{\text{loc}}{\left(r\right)}^{*}\cdot p)\}$$ (2)

$$F_{m}^{\text{dipole}}=\frac{1}{2}\mu_{0}\;\text{Re}\;\{\nabla H^{\text{loc}}{\left(r\right)}^{*}\cdot m\}\;$$ (3)

respectively. We recall that the PiFM uses a gold-coated probe tip that commonly detects the electric dipolar force exerted on an electric dipole (the nanoprobe of the PiFM) resulting from two closely spaced interacting photoinduced electric dipoles given by Eq. 2 (*23*). Therefore, in the PiFM system, as proven in (*23*), the longitudinal electric dipolar force is related to the incident transversal electric field E*_t_* as $F_{p,z}^{\text{dipole}}\propto {|\alpha^{e}E_{t}|}^{2}$, where α^e^ is the electric dipolar polarizability of the nanoprobe. As a fundamental conclusion, in a PiFM system, the electric dipolar force is directed along the beam axis, and it is directly proportional to the incident electric field intensity via the proportionality constant of the electric polarizability of the nanoprobe.

Instead, the basic principle of photoinduced magnetic force measurement is to relate the incident magnetic field (oscillating at optical frequency) to the measured longitudinal force. To understand this principle, let us consider the time-averaged magnetic dipolar force $F_{m}^{\text{dipole}}$ given by Eq. 3. We recall that the local magnetic field in Eq. 3 acting on the nanoprobe is composed of two field terms: One is the incident field, and the other is the field generated by the presence of the substrate. However, the optical magnetic force should provide useful information about the excitation field only; therefore, we equivalently represent the effect of interaction fields into the incident field following the derivation in the Supplementary Materials. As a result, the longitudinal magnetic dipolar force acting on the magnetic nanoprobe is related to the incident longitudinal magnetic field $H_{z}^{\text{inc}}$ as

$$F_{m,z}^{\text{dipole}}\propto {|\alpha_{\text{tip}}^{m}H_{z}^{\text{inc}}|}^{2}$$ (4)

where $\alpha_{\text{tip}}^{m}$ is the dipolar magnetic polarizability of the magnetic nanoprobe.

Equation 4 shows that the magnetic dipolar force along the longitudinal beam direction is proportional to the longitudinal incident magnetic field intensity via the magnetic polarizability of the magnetic nanoprobe. In accordance of the linearly proportional relationship between the electric and magnetic dipolar forces and the incident field, here, we name the electric dipolar force and magnetic dipolar force as “electric force” and “magnetic force,” respectively.

**Fig. 3.**
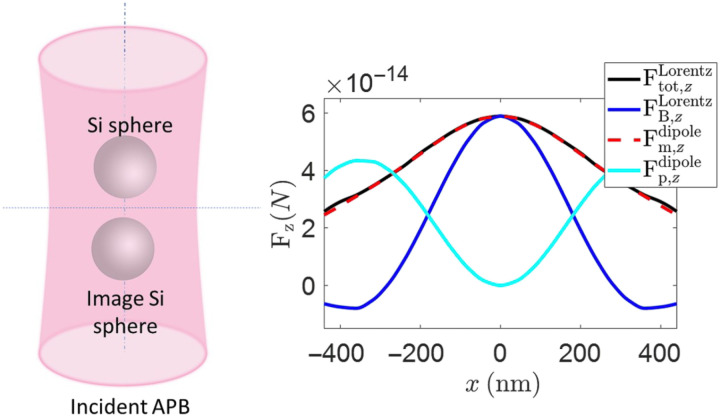
Comparison of time-averaged optical forces exerted on the spherical Si probe. These forces are in the system of two closely spaced probes shown in Fig. 2A, under APB illumination from the bottom. The abscissa represents the displacement of both spheres from the APB axis. The optical wavelength is 610 nm, corresponding to the magnetic Mie resonance. The curves represent results evaluated with the accurate total Lorentz force density in eq. S1 and the dipole approximation formulation in Eqs. 2 and 3. See also eq. S3. This result shows that $F_{\text{tot},z}^{\text{Lorentz}}\approx F_{m,z}^{\text{dipole}}$, for any displacement. See the Supplementary Materials for the incident beam and the geometrical parameters.

Next, we demonstrate the overall dominant contribution of the magnetic dipolar force to the total optical force exerted on the magnetic nanoprobe. To address this point, we have included a rigorous analytical and numerical derivation in the Supplementary Materials. We demonstrate that a dielectric Mie scatterer can be reasonably considered as a dominant magnetic dipole when the magnetic resonance is excited by an incident APB. We show that at the magnetic resonance (which we refer to as “on-state”), the Si truncated cone acts as a magnetic nanoprobe while scanning within the proximity of the incident APB. We demonstrate that the longitudinal component of the total, time-averaged optical force $F_{\text{tot},z}^{\text{Lorentz}}$ exerted on the scanning nanoprobe is almost entirely contributed by the longitudinal component of the time-averaged magnetic dipolar force defined as

$$F_{m,z}^{\text{dipole}}=\frac{1}{2}\mu_{0}\text{Re}{\left({\left(\nabla H^{\text{loc}}(r)\right)}^{*}\cdot m\right)}_{z}$$ (5)

i.e., we state that $F_{\text{tot},z}^{\text{Lorentz}}\approx F_{m,z}^{\text{dipole}}$. For convenience, we study a system of two closely spaced spheres as two Mie scatterers shown in Fig. 2A, which represents a simple physical model for the interactive system of the nanoprobe and the substrate. In Fig. 3, we show the total optical force $F_{\text{tot},z}^{\text{Lorentz}}$ exerted on the nanoprobe sphere when the two spheres are displaced from the APB axis (the derivation details are appended in the Supplementary Materials). We have also included three more curves in the figure, representing $F_{B,z}^{\text{Lorentz}}=\;\frac{1}{2}\text{Re}{\left(\int_{V}J_{\text{ind}}\times B^{*}\mathrm{dv}\right)}_{z}$, $F_{p,z}^{\text{dipole}}=\frac{1}{2}\text{Re}{\left({\left(\nabla E^{\text{loc}}(r)\right)}^{*}\cdot p\right)}_{z}$, and $F_{m,z}^{\text{dipole}}$ to corroborate our most important conclusion.

The main observation is that the total optical Lorentz force $F_{\text{tot},z}^{\text{Lorentz}}$ is equal to the magnetic dipolar force $F_{m,z}^{\text{dipole}}$ with very good accuracy. Note that the contribution of the $F_{B,z}^{\text{Lorentz}}$ term, the ∫*_V_***J**_ind_ × **B**^∗^ term in the Lorentz formula, which includes the effect of the current density **J**_ind_ that creates the artificial magnetization in the Mie resonator, does not represent the main contribution to the total optical force. Also, the term $F_{p,z}^{\text{dipole}}$ does not correlate with the observed total optical force. Therefore, we conclude that the photoinduced magnetic dipolar force $F_{m,z}^{\text{dipole}}$ is the main contributor to the total optical force $F_{\text{tot},z}^{\text{Lorentz}}$.

Next, as a comparison, we investigate the force on a photoinduced magnetic nanoprobe when the operating optical wavelength is off the magnetic resonance (referred to as the “off-state”) to show the importance of a proper selection of optical frequency in the design. As shown in the Supplementary Materials (see fig. S4), when the nanoprobe is nonmagnetic, i.e., it is in the off-state, the total optical force exerted on the dielectric nanoprobe includes nontrivial contributions from both electric and magnetic dipolar forces, and hence, the Lorentz force can neither be attributed to the magnetic nor electric dipoles.

**Fig. 4.**
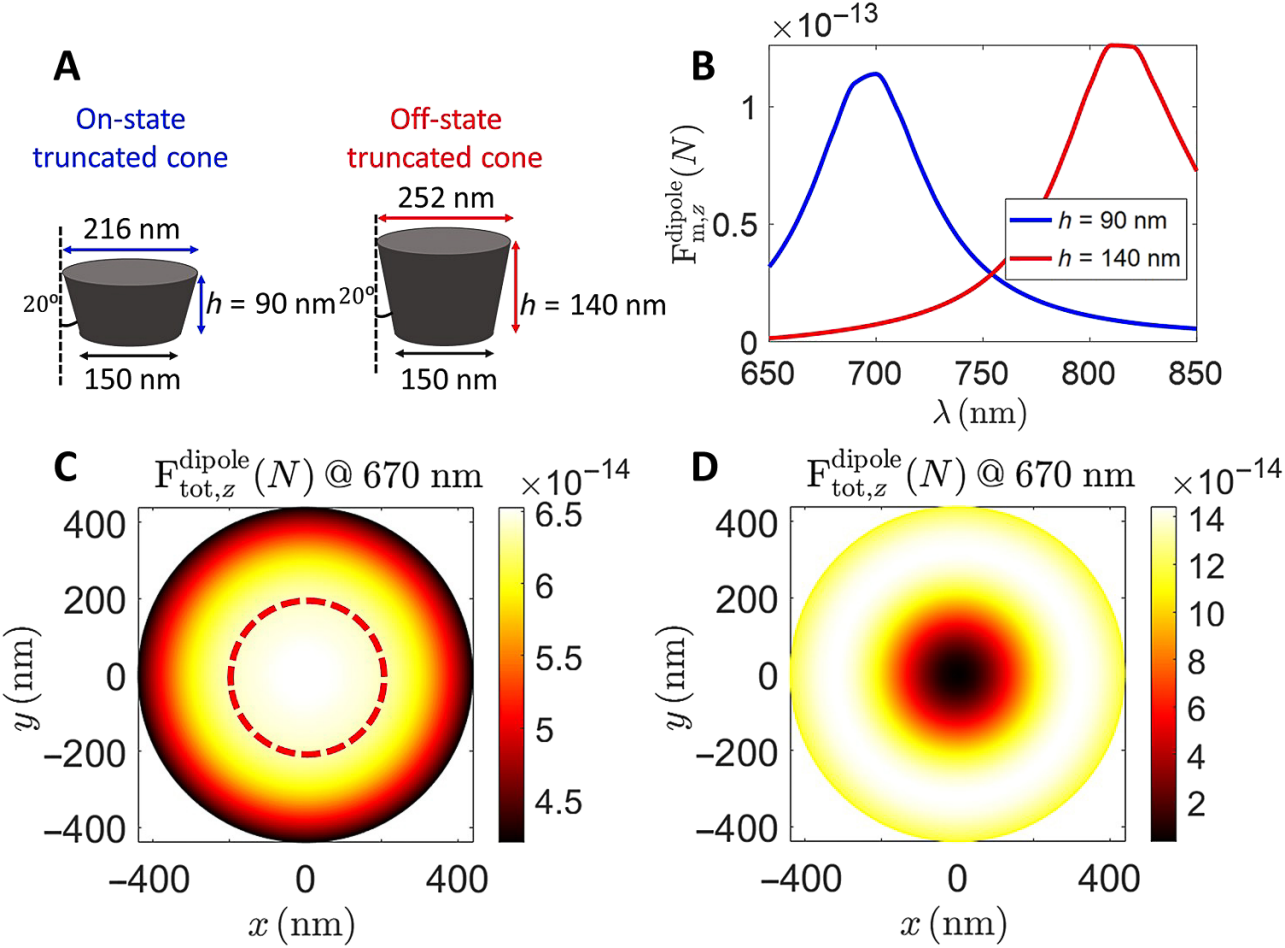
Simulation of the force profiles acquired by the on/off-state probes. (**A**) Designed on-state (left) and off-state (right) Si truncated cones with height of 90 and 140 nm, respectively, when operating at 670 nm. The side tilt angle and bottom diameter for both structures are 20° and 150 nm, respectively. (**B**) Simulated magnetic force spectrum of the on-state probe (blue) and off-state probe (red), 5 nm above a glass substrate, under APB illumination. The on-state probe generates much larger magnetic force compared to off-state probe at 670 nm. (**C** and **D**) Simulated force profiles in the transverse (*x*-*y*) plane for the on-state probe and off-state probe, respectively, operating at 670 nm. The simulations have been performed with a focused APB of incident power of 150 μW with a minimum beam waist parameter *w*_0_ = 0.7λ. The red dotted circle in (C) indicates the area of dominant magnetic force (contributing more than 90% to the total photoinduced force).

As a result, using a probe in the on-state, the photoinduced magnetic force measurement can map the incident magnetic field with superresolution via the overall optical force detection mechanism. Under APB illumination, we have theoretically shown that a magnetically polarizable nanoprobe can detect the photoinduced magnetic force of light, hence detecting the optical magnetic nearfield. This result is analogous to what was achieved in a previous work (*23*) that demonstrated that an electric nanoprobe such as a gold-coated tip could detect the photoinduced electric force and hence detect the electric nearfield (*23*). As a result, when an electric or magnetic nanoprobe raster scans a tightly focused APB, the measured force maps should exhibit the signature of the APB electric and magnetic profiles, i.e., donut and solid-center circular shapes, respectively. For the purpose of comparison and demonstration, we design the on- and off-state Si truncated cone samples shown in Fig. 4A, and we numerically analyze these states with the finite element method (*33*) implemented in the commercial simulation software COMSOL Multiphysics (*34*).

Considering the chosen laser wavelength of 670 nm, the truncated Si cone is at its on-state and at its off-state when its height is equal to 90 and 140 nm, respectively, while in both cases, the bottom (shorter) diameter is 150 nm, and the side tilt angle is 20°. As shown in Fig. 4B, the simulated magnetic force spectrum for the truncated cone placed 5 nm above the top of the glass substrate shows the unique strong force feature of on-state conditions at 670 nm. The off-state probe exhibits a much smaller magnetic dipolar force at 670 nm, and it exhibits a peak at a longer wavelength of 810 nm. The magnetic dipolar optical force exerted on the Si truncated cones in Fig. 4A has been evaluated for the truncated Si nanoprobes 5 nm above the glass substrate using Eq. 5. In the calculations, we have assumed that the incident APB light is focused on the top surface of the substrate. The incident APB has an azimuthal electric field distribution (*23*, *31*) E=φ^ (V/π)(2ρ/w2)e−ζ(ρ/w)2e−2i tan−1(z/zR)eikz, where $w=w_{0}\sqrt{1+{\left(z/z_{R}\right)}^{2}}$, ζ = 1 − *iz*/*z_R_*, $z_{R}=\pi w_{0}^{2}/\lambda$, ρ is the radial distance from the beam axis, and *w*_0_ is the minimum beam waist that is chosen to be 0.7λ, with λ being the operating wavelength. Moreover, the amplitude *V* is determined from the incident power *P*_inc_ = ∣*V*∣^2^[1 − 1/(2(π*w*_0_/λ)^2^)]/(2η_0_), where η_0_ is the free-space wave impedance. The incident power is assumed to be 150 μW. The longitudinal component of the magnetic field *H*_z_ is derived from the expression of the electric field using the Maxwell-Faraday equation **H =** ∇ × **E**/(*i*ωμ_0_), leading to *H_z_* = *A*_0_[1 − (ρ/*w*)^2^ζ]e^−ζ(ρ/*w*)^2^^e^−2*i* tan^−1^(*z*/*z_R_*)^e*^ikz^* with $A_{0}=-(V/\sqrt{\pi })[4i/(w^{2}{\omega \mu }_{0})]$. The expressions for the azimuthal electric and longitudinal magnetic fields of an APB clearly indicate a vanishing value of the electric field and a nonvanishing value of the magnetic field at the beam center.

In Fig. 4 (C and D), we show the total longitudinal force map relative to the nanoprobe (i.e., the truncated Si cone) lateral scan, perpendicular to the APB axis at 670-nm wavelength. The time-averaged longitudinal force on the truncated Si cone over the substrate has been numerically computed using the *z* component of the total optical force in Eq. 1. As illustrated in Fig. 4 (C and D), the simulated force profiles for the on- and off-state nanoprobes exhibit a bright center circular spot and a donut shape, respectively, in accordance with the magnetic and electric field profiles of the APB, respectively. This comparison shows the unique feature of the magnetic and electric fields of the APB for enabling magnetic field detection. In particular, we recognize the existence of a dominant magnetic force region inside the red dotted circle in Fig. 4C, where the magnetic force contributes more than 90% to the total photoinduced force. The derivation details are provided in the Supplementary Materials.

### Experimental demonstration

On the basis of the design of the on- and off-state nanoprobes made of truncated Si cones, we accordingly fabricated them, and we also used a blunt Si tip and a sharp Si tip as control groups. The incident APB has the wavelength of 670 nm and is focused by a bottom-illuminated oil immersion lens through the glass slip (substrate) onto the nanoprobes, as depicted in Fig. 2. The incident power into the microscope system is about 150 μW. The tightly focused APB on the front surface of the glass coverslip has a diameter of approximately 500 nm. Further details on the fabrication, the experimental optical setup, and characterization can be found in the Supplementary Materials. The spatial resolution supported by the tips is roughly determined by the diameter of the apex, which, for the on- and off-state probes, is approximately 200 nm, sufficient to resolve the spatial structure of the incident field.

**Fig. 5.**
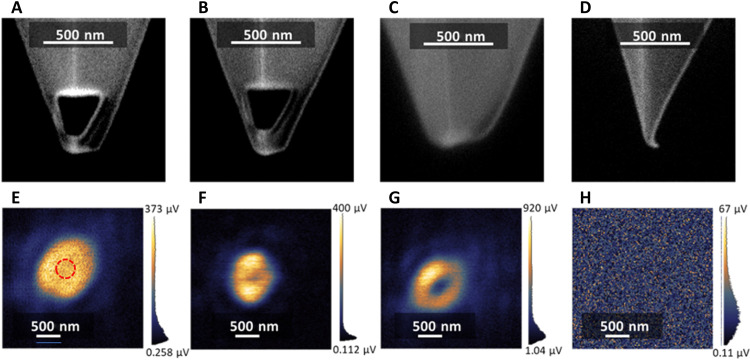
Experiment measurement of the force profiles with the corresponding probes. (**A** to **D**) FIB images of the on-state Si truncated cone probe (A), off-state Si truncated cone probe (B), blunt Si probe (C), and sharp Si probe (D). (**E** to **H**) Corresponding measured force maps upon APB illumination from the bottom of the glass slip using the on-state Si truncated cone probe (E), off-state Si truncated cone probe (F), blunt Si probe (G), and sharp Si probe (H). The on-state probe (A) measures the solid-center circular spot (E) typical of the APB magnetic field. The red dotted circle in (E) indicates the area with dominant magnetic force (contributing more than 90% to the total photoinduced force).

Figure 5 shows the experimental results: One can clearly see that the on-state nanoprobe (the one that is experiencing a dominant photoinduced magnetic dipole at 670 nm) produces a solid-center circular spot force map, as shown in Fig. 5E. In contrast, the off-state probe produces a donut-type circular-spot force map, as shown in Fig. 5F, whereas the blunt Si probe tip produces a clear donut-shape circular-spot force map, as shown in Fig. 5G, and the unmodified sharp Si probe tip produces no detectable force map, as shown in Fig. 5H. The exclusive magnetic force is evident at the bright center of the acquired force map by the on-state probe, which is demonstrated both in experiment and simulation. Similarly, we have labeled the dominant magnetic force region with the red dotted circle in Fig. 5E, in which, according to the theoretical analysis, the on-resonance magnetic force contributes more than 90% to the total photoinduced force.

Details on the physical properties of the blunt and sharp Si tip control groups in Fig. 5 (C and D) are reported in the Supplementary Materials. Here, we summarize that without a proper nanostructure at the tip end to support optical magnetic resonance, both the blunt and sharp Si tips exhibit a very weak magnetic response and cannot detect the magnetic force of light.

Note that the actual optical magnetic resonance wavelength of the fabricated on-state probe may differ slightly from the simulated model. Nonetheless, given the broad bandwidth of the magnetic Mie resonance, such a shift has a relatively minor effect on the probe’s expected magnetic response. In addition, because the electric response is suppressed on axis under APB illumination, the measured force in the red dotted circle in Fig. 5E is still expected to be predominantly magnetic. Note also that, given the width of the Mie resonance, the off-state probe is expected to exhibit a small but nonzero magnetic contribution on axis. The latter explains the partial in-filling of the “donut hole” in Fig. 5F relative to the clearer ring structure seen in Fig. 5G.

In addition, for the blunt tip, the effective tip apex resembles a truncated cone structure with a substantial width, where an effective electric dipole is created inside the structure and hence detects the electric force of light. Therefore, the force map assumes the donut shape in Fig. 5G, as already discussed in earlier experiments based on electric dipole–electric dipole interaction (*23*, *24*, *35*). A similar mechanism is also present for the sharp tip; however, in this case, the photoinduced force due to the effective electric dipole is much weaker. A simple explanation for the much weaker force for the sharp tip can be found in the Supplementary Materials. Here, we also emphasize that the exclusive and direct magnetic force acquisition from the on-state probe is only found in the center area of the acquired force map, i.e., in the vicinity of the axis of the incident APB. This acquisition is essentially dependent on the magnetic dominance of the local field. While the magnetic dominance is weakened at the off-axis location, the electric field may contribute a nontrivial part to the total optical force exerted on the probe, because the material of the probe, i.e., Si, has a natural response to the electric field as indicated for the blunt tip.

The comparison in the measured force maps obtained with different probes in Fig. 5 provides an important demonstration for the proposed magnetic field detection mechanism. From the on-state nanoprobe to the off-state nanoprobe to the blunt tip nanoprobe, the measured force maps transition from the solid-center circular shape to the donut shape. This observation is in accordance with the theoretical analysis that the photoinduced magnetic dipole contribution gradually decreases in strength for these nanoprobes. As a result, the center area of the force map given by Fig. 5E represents the target magnetic field intensity profile of the incident APB.

## DISCUSSION

We provide an overall analysis and evaluation of the results. First, we observe that the measured force maps in Fig. 5 (E and F) exhibit some asymmetry. According to a previous study of a related phenomenon (*23*), this asymmetry is caused predominantly by the anisotropy of the probe (*23*). Because the original commercial Si tip has ridges, it is naturally anisotropic. Also, the focused ion beam (FIB) fabrication of the tip will induce an arbitrary deformation, thus inevitably making it anisotropic. We expect that using a more symmetric nanoprobe would greatly improve the symmetry of the measurement, as demonstrated by previous research, which indicated that the use of a spherical gold particle can remedy the measured asymmetry by a gold-coated tip (*35*).

Second, we discuss the SNR and the resolution of the measurement. Here, we define the SNR of the force map as the maximum force amplitude divided by the average background amplitude (the force amplitude is represented in microvolts measured by the quadrant photodetectors, as shown in the scale bars). Figure 5 (E to G) reveals an SNR of 18.6, 24.4, and 14.4, respectively. In addition, we define the “magnetic SNR” as the force amplitude at the center of the force profile in Fig. 5E divided by the background noise, which gives the magnetic SNR of about 15. We note that the incident power into the microscope is around 150 μW, and we observe that the measured magnetic SNR is several times higher than the typical SNR in many conventional nearfield scanning optical microscopy or optical force measurements of structured light at the subwavelength scale (*20*, *36*, *37*).

Because the incident APB has no substantial subwavelength structure, it is not suitable for defining the measurement resolution through the measured force map. However, following the basic concepts of force microscopy, resolution is determined by the size of the nanoprobe (*24*, *25*, *38*). As a result, we can estimate the resolution in the force map shown in Fig. 5 (E and G) to be approximately the size of the probe apex as shown in Fig. 5 (A to C), i.e., about 200 nm. It is noteworthy that the resolution of each customized probe, determined by the effective size of the probe apex, can be different. Therefore, the measured force maps exhibit different overall sizes for the same incident APB. However, this difference will not affect our goal of direct and exclusive magnetic force detection because this magnetic force is only present at the center area of the APB.

The size of the photoinduced magnetic nanoprobe also depends on the wavelength of the chosen magnetic resonance. Depending on the potential improvement of design and fabrication techniques, it is possible to make an effectively smaller nanoprobe to replace the current truncated cone structure. On the basis of Mie scattering principles, an Si magnetic Mie scatterer may have the size of about one-fourth of the excitation wavelength. Furthermore, considering the bandwidth of the magnetic resonance as shown in Fig. 4B, the minimum size of the Mie scatterer can reach sub–100 nm for it to span the visible range of the spectrum, underlining that higher spatial resolution can be achieved.

Next, we have demonstrated the performance of optical magnetic force detection using an Si truncated cone as a nanoprobe tip that is able to directly detect the local magnetic field when using an APB illumination. The structured light–induced magnetic excitation of the nanoprobe enables direct magnetic force detection while decoupling the electric field interaction. The azimuthal symmetry of the Si nanoprobe and its illumination beam is a key feature. The beam’s magnetic field with axial symmetry induces the electric displacement current with azimuthal symmetry in the nanoprobe, which is equivalent to the magnetic dipole. The proper definition of magnetic dipoles is a circulating current, which allows us to recognize that the magnetic dipolar force term $F_{m,z}^{\text{dipole}}$ defined previously is the dominant contributor to the total force. In other words, although Si has a high material permittivity, the nanoprobe has no electric response because its local electric field is vanishing on the APB axis, where the magnetic field is dominant.

A previous work has achieved the sensing of forces including some of magnetic nature through the interaction between a dielectric probe and a resonating nanostructure with two holes on a glass slide that expresses well-oriented magnetic field lines (*20*). However, we emphasize that that method, due to the lack of azimuthal symmetry of the used excitation and resonating structure, cannot suppress the electric response of the probe and is thus unable to detect the magnetic force exclusively. Our current work elaborates on the definition of the gradient magnetic force and how it is related to the general Lorentz force, which explains the various force components that contribute to the total force and highlights the dominant contribution of the magnetic force.

We have demonstrated that the total optical force exerted on the magnetic polarizable tip in a PiFM configuration is dominated by the magnetic dipolar force. We have used an Si truncated cone as the photoinduced magnetic nanoprobe mounted in a force microscope with axis-aligned APB illumination. The mutual azimuthal symmetry of the incident APB and the nanoprobe enables exclusive magnetic excitation of the photoinduced nanoprobe. The measured photoinduced magnetic force is proportional to the local magnetic field detected by a scanning nanoprobe near the center of the focused APB. Consequently, we are able to directly detect the magnetic field intensity profile of an incident APB.

Therefore, in this work we have set an important milestone to conceive a photoinduced magnetic force instrument that is able to directly and selectively detect the magnetic field of light at the nanoscale. We underscore that the detection of the magnetic field of light is one essential step toward the ultimate understanding of the complete electromagnetic properties of matter. The capabilities of the photoinduced magnetic force instrument can benefit emerging research areas in nanophotonics, such as the characterization and manipulation of the optomagnetic properties in nanostructures (*39*).

## MATERIALS AND METHODS

The Si truncated cone probe is made on the basis of the commercial Si atomic force microscopy probe from Nanosensors (PPP-NCHR), which has the nominal mechanical resonance frequency at 330 kHz. We use the FIB lithography to drill a hole from the side of the Si probe and remove its sharp tip by using the Quanta 3D FEG Dual Beam scanning electron microscope from FEI Thermo Fisher Scientific. The Si material of the commercial probe is a single crystalline silicon doped with antimony (N doped). According to the vendor, the permittivity of this Si is well characterized and used on the basis of the previous literature (*40*).

We use the continuous-wave single-mode fiber pigtailed laser diode with 670-nm wavelength from Thorlabs Inc. as the light source. We use the polarization converter from ARCoptix to generate the APB.

We perform all the photoinduced force measurements by using the commercial PiFM VistaScope from Molecular Vista Inc. Inside the VistaScope, the incident APB is sharply focused by a high–numerical aperture oil-immersed objective lens from Olympus. We optimize various parameters/settings of the VistaScope to improve SNR measurement results. The minimum detectable force of the VistaScope can reach subpiconewtons in range (*41*).

## Supplementary Materials

### This PDF file includes:

Supplementary Text Figs. S1 to S6 References
