# Supplementary material for: Direct detection of photoinduced magnetic force at the nanoscale reveals magnetic nearfield of structured light
Source: Sci Adv. 2022 Nov 9;8(45):eadd0233. doi: 10.1126/sciadv.add0233 (PMC9645709; doi:10.1126/sciadv.add0233)
Supplement: Supplementary file 1 — Supplementary Text Figs. S1 to S6 References [file sciadv.add0233_sm.pdf]

Science Advances
AAAS

Supplementary Materials for

Direct detection of photoinduced magnetic force at the nanoscale reveals magnetic nearfield of structured light

Jinwei Zeng *et al.*

Corresponding author: Jinwei Zeng, zengjinwei@hust.edu.cn; Eric O. Potma, epotma@uci.edu; H. Kumar Wickramasinghe, hkwick@uci.edu; Filippo Capolino, f.capolino@uci.edu

Sci. Adv. **8**, eadd0233 (2022)
DOI: 10.1126/sciadv.add0233

This PDF file includes:

Supplementary Text
Figs. S1 to S6
References

Si probe on top of glass substrate illuminated by an APB

As the essential part of the proposed photoinduced magnetic force detection method, in this subsection we discuss a physical modeling for the Si probe on top of the glass substrate illuminated by an incident APB.

As has already been discussed in a previous work (24), a subwavelength Si nanoparticle can be considered as a pure magnetic dipole scatterer provided that a proper symmetric excitation is applied to exclusively excite its magnetic dipole moment. In that work, we have experimentally obtained the force map, based on the electric field component of light, exerted on a gold tip on top of a Si truncated cone located on a glass substrate and illuminated by an APB. Therefore, in that work we measured the electric dipolar force exerted on the scanning nanotip (that is an electric nanoprobe). We have shown that the measured force is proportional to the incident electric field intensity, and that is the reason we call it the electric force of light (23). Based on experimental results and on rigorous theoretical analysis based on the calculation of multipole moments, in (24) we have *indirectly* demonstrated (from the measurement of the electric force) that a Si disk is an effective magnetic scatterer at a specific wavelength range and under APB excitation.

Here we use the magnetic scatterer investigated in (24) as a probe of the magnetic force, and devise a new force microscopy technique which enables *direct and exclusive* acquisition of the optical force due to the magnetic field component of light rather than its electric counterpart. As noted in the main text of the manuscript, this method enables the direct measurement of magnetic field component of light, in contrast of the common PiFM system that only directly detects the electric field component of light.

Considering the dipole approximation of the Si magnetic nanoprobe of the proposed system shown in Fig. S1, the validity of which is provided in the following, the simplest scenario that mimics the interaction between the Si probe (as a dipole scatterer) and the glass substrate under APB illumination is shown in Fig. S1 (Right). First, we replace the Si magnetic nanoprobe with a Si sphere that carries a Mie “magnetic” resonance, then we replace the overall effect of the substrate with an image dipole. As a result, we consider two closely spaced Si sphere that mainly provide two coupled magnetic dipoles; one representing the probe whereas the other illustrating its image. In the following we also explore the role of the other contributions like the electric dipole and quadrupoles, demonstrating the dominance of the magnetic dipole in generating the total force on the nanoprobe. Fig. S1 illustrates these two scenarios, i.e., the real scenario of the Si probe on top of the glass substrate, and the equivalent scenario of the two identical spheres illuminated by an APB from the bottom (42).

The equivalent problem of two interacting magnetic dipolar dipoles is treated analytically and it provides a basic physical insight of the real scenario. During the whole analysis, we keep in mind that the main goal is to find the optical force exerted on the Si probe on top of the substrate when illuminated by an APB from the bottom (see Fig. S1).

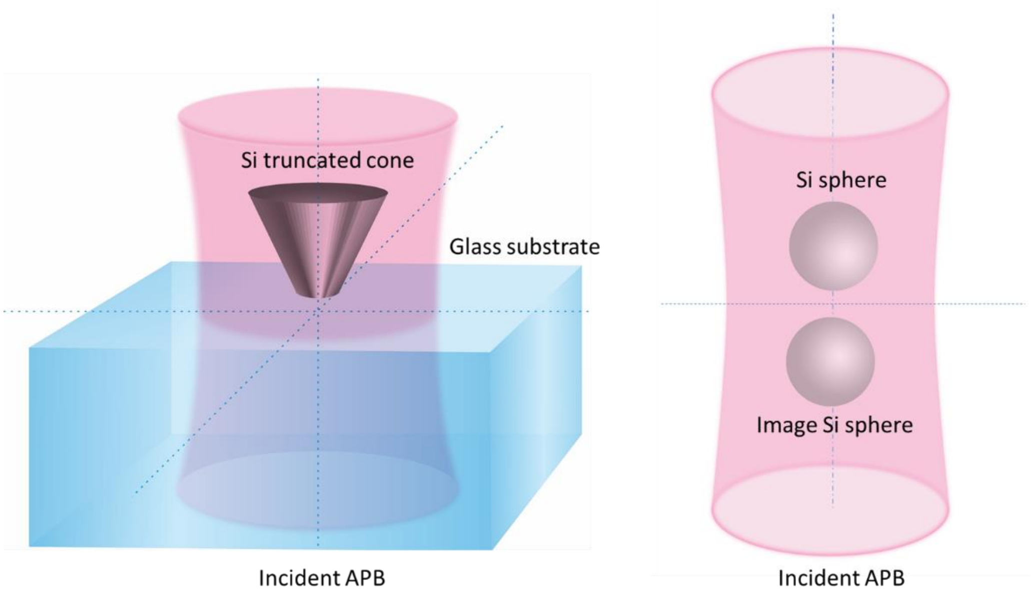

Fig. S1. Real scenario Left) and its physical modeling Right). The Si probe on the top of a glass substrate is illuminated with an APB incident from the bottom side. In the physical modeling, the truncated cone shape of the probe is modeled as a sphere shape for the convenience of analytical derivation. And the substrate is replaced by the image sphere to mimic the electromagnetic interaction between the probe and the substrate.

The Lorentz force density and the total optical force

The optical force on an object is derived from the Lorentz force density generated by a distribution of charge and current densities $\rho(\mathbf{r},t)$ and $\boldsymbol{J}(\mathbf{r},t)$, respectively. The Lorentz force *density* $\boldsymbol{f}(\mathbf{r},t)$ exerted at any position $\mathbf{r}$ is given by

$$\boldsymbol{f}(\mathbf{r},\mathrm{t}) = \rho\boldsymbol{E} + \boldsymbol{J}\times\boldsymbol{B}. \tag{S1}$$

where $\boldsymbol{E}(\mathbf{r},t)$ is the electric field vector, and $\boldsymbol{B}(\mathbf{r},t)$ is the magnetic induction vector. Here all italic symbols define position and time dependent quantities. Therefore, the *total* force on the proposed object with volume V containing these charge and current distributions reads $\boldsymbol{F}_{\mathrm{tot}}^{\mathrm{Lorentz}}(t) = \int_V \boldsymbol{f}\, dv$. Since we do not consider any "impressed" (or forced) source within the magnetic nanoprobe volume in our scenario, then, the force is calculated using the induced charge and current densities, $\rho_{\mathrm{ind}}(\mathbf{r},t)$ and $\boldsymbol{J}_{\mathrm{ind}}(\mathbf{r},t)$, respectively in the magnetic nanoprobe, leading to $\boldsymbol{f} = \rho_{\mathrm{ind}}\boldsymbol{E} + \boldsymbol{J}_{\mathrm{ind}}\times\boldsymbol{B}$. The total time-average force exerted on the probe is provided by volume integration, leading to

$$\mathbf{F}_{\text{tot}}^{\text{Lorentz}} = \frac{1}{2}\text{Re}\left(\int_V \rho_{\text{ind}}\mathbf{E}^* + \mathbf{J}_{\text{ind}} \times \mathbf{B}^* dv\right) \tag{S2}$$

where non-italic fonts denote phasors, * denotes complex conjugation, and we have implicitly assumed a $\text{e}^{-i\omega t}$ time dependence.
The volumetric integral (S2) and has been numerically calculated directly using COMSOL evaluated fields, to produce the results of $\text{F}_{\text{tot},z}^{\text{Lorentz}}$ in Figs. 2, S3, and S4. The term $\text{F}_{\text{B},z}^{\text{Lorentz}}$ in Fig. 3 has been calculated in the same way, but using only the term $\mathbf{J}_{\text{ind}} \times \mathbf{B}^*$ in (S2).

Optical force and the dipole approximation

By neglecting all multipoles except the dipoles, one can model the nanoprobe as a dipolar scatterer with induced electric and magnetic dipole moments $\mathbf{p}$ and $\mathbf{m}$, respectively. The time-averaged optical force $\mathbf{F}$ exerted on the object for time harmonic fields with time dependence $\text{e}^{-i\omega t}$ is represented in terms of phasors as follows (13):

$$\mathbf{F}_{\text{tot}}^{\text{dipole}} = \frac{1}{2}\text{Re}\left\{\left(\nabla\mathbf{E}^{\text{loc}}(\mathbf{r})\right)^* \cdot \mathbf{p} + \mu_0\left(\nabla\mathbf{H}^{\text{loc}}(\mathbf{r})\right)^* \cdot \mathbf{m} - \mu_0 \frac{ck^4}{6\pi}(\mathbf{p}\times\mathbf{m}^*)\right\}, \tag{S3}$$

The i-th component of the time-averaged optical force F_i in Eq. (S3) reads,

$$\text{F}_{\text{tot},i}^{\text{dipole}} = \frac{1}{2}\text{Re}\left\{\sum_j\left[\text{p}_j\left(\partial_i \text{E}_j^{\text{loc}}\right)^* + \mu_0 \text{m}_j\left(\partial_i \text{H}_j^{\text{loc}}\right)^*\right] - \mu_0 \frac{ck^4}{6\pi}\left(\mathbf{p}\times\mathbf{m}^*\right)_i\right\} \tag{S4}$$

for $i, j = x, y, z$ Cartesian coordinates. Note that one may consider the effect of higher order multipoles and calculate the optical force exerted on the object as in Ref. (43), however, within an acceptable approximation range it is enough to only include the effect of dipole moments in our analysis, as demonstrated in our results when comparing the dipolar force to the total Lorentz force.

The system of coupled Si probe-image under APB illumination

Let us first demonstrate that the probe in the system of a coupled sphere under APB illumination is a magnetic scatterer at a specific wavelength range and under certain alignment condition. To that end, we calculate the multipoles (up to magnetic quadrupoles) of the top sphere (as the model of the probe) when it is coupled with the image sphere (that mimics the effect of the substrate). As the representation of the realistic fabricated probe, we design the probe and image spheres to have the comparable size with 92nm radius, and their magnetic resonance wavelength is around 610 nm. We prove that under a specific alignment between the two spheres and the beam, the probe sphere is exclusively a magnetic dipole scatterer. Indeed, we calculate and compare the power scattered by each multipole and show that under such an alignment the scattered power due to the magnetic dipole of the probe sphere is dominant. Accordingly, we consider only the first four multipoles, i.e., electric and magnetic dipole as well as quadrupole moments $\mathbf{p}$, $\mathbf{m}$, $\bar{\bar{\mathbf{Q}}}^{\mathbf{e}}$, and $\bar{\bar{\mathbf{Q}}}^{\mathbf{m}}$, respectively, which are enough for our analysis proof due to the small size of the scatterer

within the frequency band of interest (i.e., $2r \sim 0.3\lambda_r$, where $2r = 184\text{nm}$ is the diameter of the Si sphere and $\lambda_r = 610\text{nm}$ is the magnetic resonance wavelength for this radius). The electric and magnetic dipole moments $\mathbf{p}$ and $\mathbf{m}$ are two vectors whereas the electric and magnetic quadrupole moments $\bar{\bar{\mathbf{Q}}}^{\mathbf{e}}$ and $\bar{\bar{\mathbf{Q}}}^{\mathbf{m}}$ are two tensors of second rank. The components of each moment read (42, 44, 45):

$$\begin{aligned} &\mathrm{p}_\alpha = \int_V r_\alpha\, \rho(\mathbf{r})\, dv, \qquad \mathrm{m}_\alpha = \frac{1}{2}\int_V \left[\mathbf{r}\times\mathbf{J}(\mathbf{r})\right]_\alpha dv, \\ &\mathrm{Q}^{\mathrm{e}}_{\alpha\beta} = \int_V \left(3r_\alpha r_\beta - r^2\delta_{\alpha\beta}\right)\rho(\mathbf{r})\, dv, \qquad \mathrm{Q}^{\mathrm{m}}_{\alpha\beta} = \frac{1}{3}\int_V \left(\left[\mathbf{r}\times\mathbf{J}(\mathbf{r})\right]_\alpha r_\beta + \left[\mathbf{r}\times\mathbf{J}(\mathbf{r})\right]_\beta r_\alpha\right) dv, \end{aligned} \tag{S5}$$

respectively. Here, in Cartesian coordinates, α and β are x, y and z coordinates and such indices are used to represent Cartesian components of vectors and tensors, whereas $\mathbf{r} = x\hat{\mathbf{x}} + y\hat{\mathbf{y}} + z\hat{\mathbf{z}}$ is the position vector and $r = \sqrt{x^2 + y^2 + z^2}$. Moreover, $\delta_{\alpha\beta}$ is the Kronecker delta, ρ and $\mathbf{J}$ are, respectively, the charge and current density distributions over the scatterer volume *V*, and the integrations are taken over the entire volume of the scatterer. It can be shown that the scattered power P_{scat} due to these multipoles reads (42, 46):

$$P_{\text{scat}} = \frac{\omega k^3}{12\pi}\left[\sum_\alpha \left(\frac{|\mathrm{p}_\alpha|^2}{\epsilon_0} + \mu_0 |\mathrm{m}_\alpha|^2\right) + \frac{1}{120}\sum_{\alpha,\beta}\left(\frac{|k\mathrm{Q}^{\mathrm{e}}_{\alpha\beta}|^2}{\epsilon_0} + \mu_0 |k\mathrm{Q}^{\mathrm{m}}_{\alpha\beta}|^2\right)\right]. \tag{S6}$$

Here ω and k are angular frequency and wavenumber whereas ϵ_0 and μ_0 are the free space permittivity and permeability, respectively. Fig. S2 demonstrates the contribution to the total scattered power, Eq. (S6), generated by the Si probe sphere, provided by the electric and magnetic dipoles, and by the electric and magnetic quadrupoles defined in Eq. (S5). Results are obtained using full-wave numerical calculations carried out with COMSOL Multiphysics software which is based on the finite element method (33) (FEM).

In Fig. S2 we plot the multipole contributions to the total scattered power given by Eq. (S6) for the probe sphere by moving the system of two spheres from the center of the beam axis (at *x* = 0 nm) toward the edge of the beam waist (*x* = 450 nm). The two spheres have a radius of 92nm radius nm, and the gap between them is 5 nm. In these calculations, we have used an APB with beam waist parameter $w_0 = 0.7\lambda$ and carrying a power of 150 μW coming from the bottom, with its minimum waist at *z* = 0. As it is clear from this figure, the scattering power due to the magnetic dipole is dominant when the system of two spheres is fully aligned with the axis of the excitation beam (at *x*=0). In such a scenario, the total scattered power peaks at $\lambda = 610\,\text{nm}$ which corresponds to its magnetic resonance. Interestingly, the footprint of the magnetic dipole always presents even when the nanoprobe is laterally displaced with respect to the beam axis when the scattering of the electric dipole is dominant.

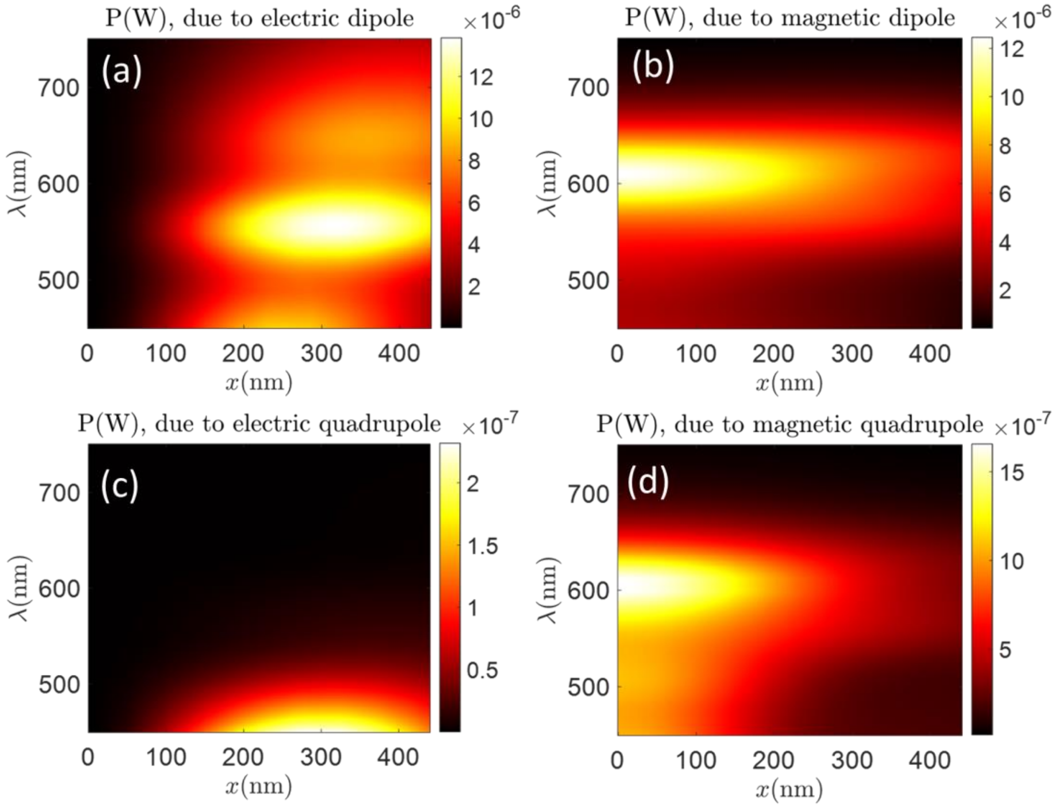

Fig. S2. Contribution of dominant multipole moments to the total scattered power. The multupole moments are generated by the top Si sphere, Eq. (S6), versus displacement x between the position of the spherical nanoprobe and the APB axis. Position $x = 0$ denotes the case when the tip and sample spheres are fully aligned with the beam axis. (a) Electric dipole; (b) magnetic dipole; (c) electric quadrupole; and (d) magnetic quadrupole of the tip in the system of two coupled spheres.

<u>The exerted force on the Si probe sphere in the presence of its image sphere under the APB illumination</u>

We have already proven in the previous section that scattering by the probe Si sphere at the presence of the image is dominated by the scattering of the magnetic dipole moment at the wavelength of interest, when illuminated by an APB. Here we calculate the exerted force on this magnetic Mie resonator integrating the Lorentz force density formulation of Eq. (S1), and demonstrate that the total force calculated using this force formulation on this magnetic resonator with a good approximation is equivalent to

$$\mathrm{F}_{\mathrm{m},i}^{\mathrm{dipole}} = \frac{1}{2}\mu_o \,\mathrm{Re}\left\{\sum_j \mathrm{m}_j \left(\partial_i \mathrm{H}_j^{\mathrm{loc}}\right)^*\right\}, \tag{S7}$$

where, ∂_i is partial derivative with respect to the i-th spatial coordinate, and $i,j=x,y,z$ are the Cartesian coordinates. This corresponds to the components of the magnetic dipole force expression

in Eq. (3). Indeed, in the main body of the paper, this force was called as the magnetic dipolar force since it only includes the interaction between the magnetic dipole moment and the magnetic field gradient. In this paper, the magnetic dipole force in Eqs. (S7) and (3) is evaluated by the product of the numerically calculated magnetic dipole as in Eq. (S5) and the numerically calculated magnetic field gradient at the center of the tip sphere, by using the finite element method simulations implemented in COMSOL Multiphysics. When this formula is applied to the Si truncated cone tip, the gradient is evaluated at the center of the cone.

Fig. S3 shows the total optical force map, calculated using (S2), exerted on the top Si *sphere* (representing the tip of the probe) and its image sphere illuminated by an APB from the bottom. The two spheres system is the same as the one considered in the previous section, i.e., the have a radius of 92nm radius nm, and the gap between them is 5 nm. The force is calculated at each position when we laterally move the Si tip and image spheres, relative to the APB illumination axis for two wavelengths: 610 nm (magnetic resonance) and 550 nm (electric resonance). As shown in this figure, the force map shows a bright center circular shape with a hot spot at the magnetic resonance of the scatterer at 610 nm, and a doughnut shape at the electric resonance (550 nm) as it is also the case in the experimental measurements discussed in the main body of the manuscript. Importantly, the force map at the magnetic resonance resembles the magnetic field distribution as discussed in the main manuscript and will be shown in a next section here. Indeed, this figure proves that the magnetic force and the magnetic field are proportional as shown in Eq. (4) in the manuscript and is proven below.

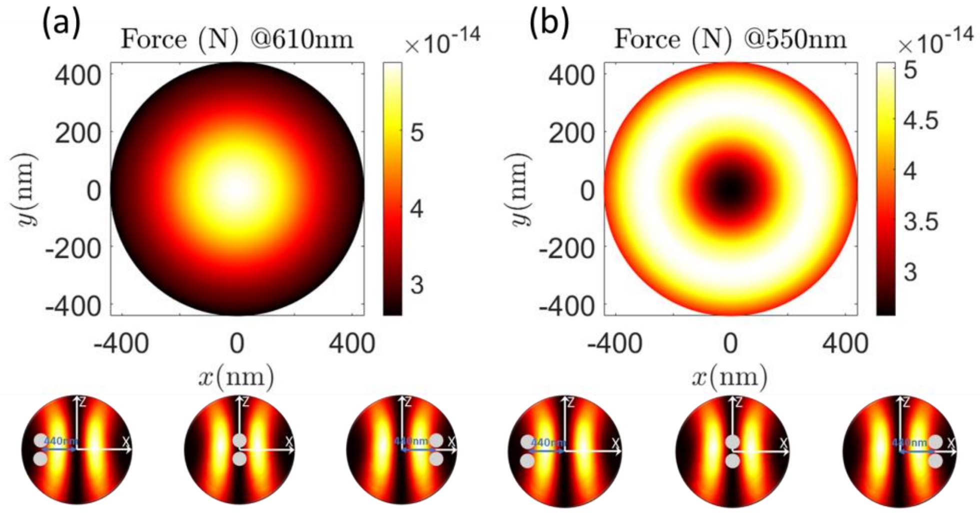

Fig. S3. Total optical force distribution exerted on the Si spherical probe. This force is in the system of probe-image spheres and APB illumination from the bottom, when the beam axis and the system of two spheres are relatively moved with respect to each other at (a) 610 nm and (b) 550 nm. The force is calculated using Eq. (S2) for an illumination power of 150 μW.

In Fig. S4 we compare the exerted force calculated from the Lorentz total force formulation in Eq. (S1) integrated over the whole top sphere with the contributions due to the electric and

magnetic dipolar forces calculated from the dipole approximation formulation in Eq. (S3). The comparison is done at two wavelengths: at 610 nm, as the *on-state* condition (on magnetic resonance), and at 550 nm, as the *off-state* condition (off magnetic resonance).

The different terms in Fig. S4 are all longitudinal force components defined as follows: the time-averaged *total* optical force $F_{tot,z}^{Lorentz}$ exerted on the nanoprobe is defined in Eq. (S2); the time-average *magnetic dipolar force* $F_{m,z}^{dipole}$ is defined in Eqs. (3) and (S7); the time-average second term in the Lorentz formula due to the magnetic field **B** is $F_{B,z}^{Lorentz} = \frac{1}{2}\mathrm{Re}\left(\int_V \mathbf{J}_{ind} \times \mathbf{B}^* dv\right)_z$; and the time-average electric dipolar force $F_{p,z}^{dipole}$ is defined in Eqs. (2). As shown in Fig. S4(a), the total force $F_{tot,z}^{Lorentz}$ exerted on the tip is in close agreement with the magnetic dipolar force $F_{m,z}^{dipole}$ in a wide range of relative positions between the beam axis and the two coupled scatterers at the magnetic resonance wavelength whereas the agreement does not hold for the off-magnetic resonant wavelength shown in Fig. S4(b). This proves that the total optical force is purely magnetic within the interested wavelength range and under this specific type of excitation scenario.

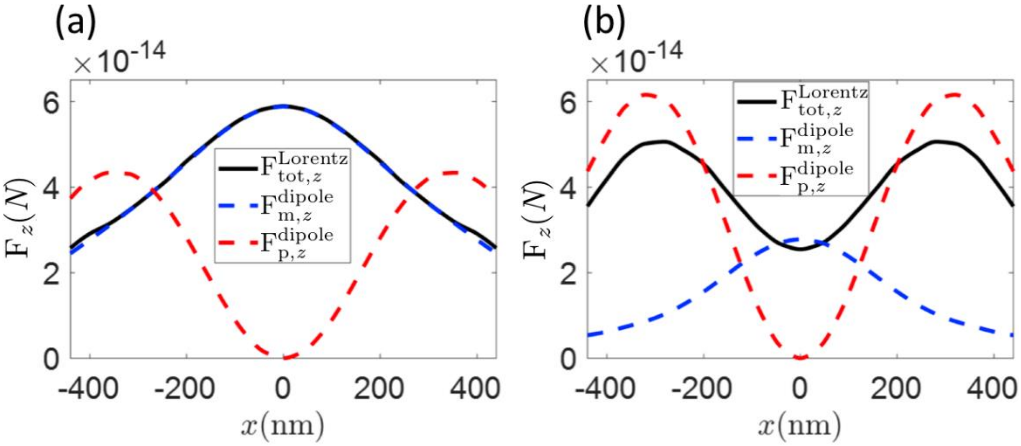

Fig. S4. Comparison of optical forces exerted on the Si *spherical* tip for the on-/off-resonance cases. The forces are calculated in the system made of tip-image spheres under APB illumination, at the magnetic resonance 610 nm (a), and off-resonance 550 nm (b). The force $F_{tot,z}^{Lorentz}$ is numerically calculated using Eq. (S2). The dipolar forces $F_{p,z}^{dipole}$ and $F_{m,z}^{dipole}$ are respectively evaluated using Eqs. (2) and (3) in the main body of the paper. The *x* coordinate represents the displacement of the two spheres with respect to the beam axis. When on-resonance, the accurate Lorentz force $F_{tot,z}^{Lorentz}$ is well represented by the magnetic dipole force $F_{m,z}^{dipole}$.

<u>Dependence of the total dipolar force on the sharp/blunt probe</u>

Given the tapered shaft of the sharp tip, we note that a stronger optical response from the tip can be expected at locations higher up the shaft, as circulating currents associated with the Mie resonance are more prominent when the shaft diameter gradually increases. For sharper tips at the chosen excitation wavelength

these currents move from the apex towards the base of the conical tip and that is why the dipole-dipole distance increases for sharper tips. A similar phenomenon has been reported in the literature (47, 48). Since the photo-induced force decreases as $1/d^4$, where d is the distance between the sample and the location in the tip material, a meaningful force contribution from the tip material higher up the shaft (larger d) is suppressed. Consequently, the overall response of the sharp Si tip is expected to be much weaker than the response of the blunt Si tip.

Derivation of Eq. (4) of the main manuscript

Referring to Eq. (S7), the local magnetic field, H_j^{loc} possesses one contribution from the incident APB beam and the other one from the scattered beam due the reflections from the substrate. Here by using the image principle (42), we replace the substrate with an image dipole, and consider its interactive effect in the field contributions from the image dipole. Therefore, the local magnetic field at the tip position reads

$$\mathbf{H}^{\text{loc}}\left(\mathbf{r}_{\text{tip}}\right) = \mathbf{H}^{\text{inc}}\left(\mathbf{r}_{\text{tip}}\right) + \mathbf{H}^{\text{scat}}_{\text{img}\to\text{tip}}\left(\mathbf{r}_{\text{tip}}\right) \tag{S8}$$

where,

$$\mathbf{H}^{\text{scat}}_{\text{img}\to\text{tip}}\left(\mathbf{r}_{\text{tip}}\right) = \underline{\mathbf{G}}(\mathbf{r}_{\text{tip}} - \mathbf{r}_{\text{img}}) \cdot \mathbf{m}_{\text{img}} \tag{S9}$$

and $\underline{\mathbf{G}}(\mathbf{r}) = G_x\hat{\mathbf{x}}\hat{\mathbf{x}} + G_y\hat{\mathbf{y}}\hat{\mathbf{y}} + G_z\hat{\mathbf{z}}\hat{\mathbf{z}}$ is the magnetic dyadic Green's function in Cartesian coordinates with $\mathbf{r}$ being the position vector at the tip/image location. Due to optically small distance between the tip and its image, the near field term in the Green's function is dominant and is approximated by (42):

$$\underline{\mathbf{G}}(\mathbf{r}) = \frac{1}{4\pi|\mathbf{r}|^3}\left(3\hat{\mathbf{r}}\hat{\mathbf{r}} - \underline{\mathbf{I}}\right) \tag{S10}$$

where $\underline{\mathbf{I}}$ is the identity tensor of rank 2 and $\hat{\mathbf{r}}$ is the unit vector from the source to the observation point. The dipole moments of the image dipole and the tip dipole are

$$\mathbf{m}_{\text{img}} = \underline{\boldsymbol{\alpha}}^m_{\text{img}} \cdot \mathbf{H}^{\text{loc}}(\mathbf{r}_{\text{img}}), \tag{S11}$$

$$\mathbf{m}_{\text{tip}} = \underline{\boldsymbol{\alpha}}^m_{\text{tip}} \cdot \mathbf{H}^{\text{loc}}(\mathbf{r}_{\text{tip}}). \tag{S12}$$

Here $\underline{\boldsymbol{\alpha}}^m_{\text{tip}}$ and $\underline{\boldsymbol{\alpha}}^m_{\text{img}}$ are the dipolar magnetic polarizability tensors of the tip and image, respectively. Similarly, the local magnetic field at the image position reads

$$\mathbf{H}^{\text{loc}}\left(\mathbf{r}_{\text{img}}\right) = \mathbf{H}^{\text{inc}}\left(\mathbf{r}_{\text{img}}\right) + \mathbf{H}^{\text{scat}}_{\text{tip}\to\text{img}}\left(\mathbf{r}_{\text{img}}\right) \tag{S13}$$

where,

$$\mathbf{H}^{\text{scat}}_{\text{tip}\to\text{img}}\left(\mathbf{r}_{\text{img}}\right) = \underline{\mathbf{G}}(\mathbf{r}_{\text{img}} - \mathbf{r}_{\text{tip}}) \cdot \mathbf{m}_{\text{tip}} \tag{S14}$$

Inserting Eq. (S14) in Eq. (S13) and then by using the resulting equation in Eq. (S11), we derive a closed-form expression for $\mathbf{m}_{\text{img}}$ which is eventually used to obtain $\mathbf{H}^{\text{loc}}\left(\mathbf{r}_{\text{tip}}\right)$ from Eqs.

(S8) and (S9). Therefore, the longitudinal component of the optical force at the tip position from Eq. (S7) reads

$$\mathrm{F}_{\mathrm{m},z}^{\mathrm{dipole}} = \frac{1}{2}\mu_o \,\mathrm{Re}\left\{ \mathrm{m}_{\mathrm{tip},x}\left(\partial_z \mathrm{H}_x^{\mathrm{loc}}\left(\mathbf{r}_{\mathrm{tip}}\right)\right)^* + \mathrm{m}_{\mathrm{tip},y}\left(\partial_z \mathrm{H}_y^{\mathrm{loc}}\left(\mathbf{r}_{\mathrm{tip}}\right)\right)^* + \mathrm{m}_{\mathrm{tip},z}\left(\partial_z \mathrm{H}_z^{\mathrm{loc}}\left(\mathbf{r}_{\mathrm{tip}}\right)\right)^* \right\} \quad \text{(S15)}$$

Finally, by using Eq. (S12) in the above equation, we obtain

$$\begin{aligned}\mathrm{F}_{\mathrm{m},z}^{\mathrm{dipole}} = \frac{3}{8\pi d^4}\mathrm{Re}\sum_j \Big\{ &\left(\alpha_{\mathrm{tip},xj}^{\mathrm{m}} \mathrm{H}_j^{\mathrm{inc}}\left(\mathbf{r}_{\mathrm{tip}}\right)\right)\left(\alpha_{\mathrm{img},xj}^{\mathrm{m}} \mathrm{H}_j^{\mathrm{inc}}\left(\mathbf{r}_{\mathrm{img}}\right)\right)^* + \left(\alpha_{\mathrm{tip},yj}^{\mathrm{m}} \mathrm{H}_j^{\mathrm{inc}}\left(\mathbf{r}_{\mathrm{tip}}\right)\right)\left(\alpha_{\mathrm{img},yj}^{\mathrm{m}} \mathrm{H}_j^{\mathrm{inc}}\left(\mathbf{r}_{\mathrm{img}}\right)\right)^* \\ &-2\left(\alpha_{\mathrm{tip},zj}^{\mathrm{m}} \mathrm{H}_j^{\mathrm{inc}}\left(\mathbf{r}_{\mathrm{tip}}\right)\right)\left(\alpha_{\mathrm{img},zj}^{\mathrm{m}} \mathrm{H}_j^{\mathrm{inc}}\left(\mathbf{r}_{\mathrm{img}}\right)\right)^* \Big\}\end{aligned} \quad \text{(S16)}$$

where, $\alpha_{\mathrm{tip},ij}^{\mathrm{m}}$ and $\alpha_{\mathrm{img},ij}^{\mathrm{m}}$ $(i, j = x, y, z)$ are the magnetic polarizability components of the tip and the image dipole, respectively, in Cartesian coordinates and d is the vertical distance between the tip and the image dipole. In obtaining Eq. (S16), we considered that the tip and its image are positioned along the z-axis. Moreover, in determining the local magnetic fields, we neglected terms containing incident fields compared to terms containing the gradient of scattered fields since their values are much smaller in the near zone of a scatterer. Besides, we have ignored all the terms containing polarizability power orders higher than second and since the dipole and its image are optically very close, we have neglected the terms containing $G = 1/\left(4\pi|z|^3\right)$ compared to those containing $\partial G/\partial z = -3/\left(4\pi|z|^4\right)$.

Next, if the phase difference of the incident beam between the tip and its image is neglected due to their deep subwavelength distance, we can assume that $\mathbf{H}^{\mathrm{inc}}\left(\mathbf{r}_{\mathrm{tip}}\right) \approx \mathbf{H}^{\mathrm{inc}}\left(\mathbf{r}_{\mathrm{img}}\right) = \mathbf{H}^{\mathrm{inc}}$ and the force reduces to

$$\begin{aligned}\mathrm{F}_{\mathrm{m},z}^{\mathrm{dipole}} = \frac{3}{8\pi d^4}\mathrm{Re}\sum_j \Big\{ &\left(\alpha_{\mathrm{tip},xj}^{\mathrm{m}} \mathrm{H}_j^{\mathrm{inc}}\right)\left(\alpha_{\mathrm{img},xj}^{\mathrm{m}} \mathrm{H}_j^{\mathrm{inc}}\right)^* + \left(\alpha_{\mathrm{tip},yj}^{\mathrm{m}} \mathrm{H}_j^{\mathrm{inc}}\right)\left(\alpha_{\mathrm{img},yj}^{\mathrm{m}} \mathrm{H}_j^{\mathrm{inc}}\right)^* \\ &-2\left(\alpha_{\mathrm{tip},zj}^{\mathrm{m}} \mathrm{H}_j^{\mathrm{inc}}\right)\left(\alpha_{\mathrm{img},zj}^{\mathrm{m}} \mathrm{H}_j^{\mathrm{inc}}\right)^* \Big\}\end{aligned} \quad \text{(S17)}$$

If one considers a reference system such that the polarizability tensors are diagonal, i.e., $\alpha_{\mathrm{img},ij}^{\mathrm{m}} = \alpha_{\mathrm{tip},ij}^{\mathrm{m}} = 0; \quad i \neq j$, and also assume an azimuthally symmetric scatterer, i.e., $\alpha_{\mathrm{tip},xx}^{\mathrm{m}} = \alpha_{\mathrm{tip},yy}^{\mathrm{m}} = \alpha_{\mathrm{tip},zz}^{\mathrm{m}} = \alpha_{\mathrm{tip}}^{\mathrm{m}}$ and $\alpha_{\mathrm{img},xx}^{\mathrm{m}} = \alpha_{\mathrm{img},yy}^{\mathrm{m}} = \alpha_{\mathrm{img},zz}^{\mathrm{m}} = \alpha_{\mathrm{img}}^{\mathrm{m}}$, the time-averaged optical force reads

$$F_{m,z}^{\text{dipole}} = \frac{3}{8\pi d^4} \text{Re}\left\{ \left(\alpha_{\text{tip}}^{\text{m}} H_x^{\text{inc}}\right)\left(\alpha_{\text{img}}^{m} H_x^{\text{inc}}\right)^* + \left(\alpha_{\text{tip}}^{\text{m}} H_y^{\text{inc}}\right)\left(\alpha_{\text{img}}^{\text{m}} H_y^{\text{inc}}\right)^* \right.$$
$$\left. -2\left(\alpha_{\text{tip}}^{\text{m}} H_z^{\text{inc}}\right)\left(\alpha_{\text{img}}^{\text{m}} H_z^{\text{inc}}\right)^* \right\} \tag{S18}$$

For the case of Azimuthally polarized beam we have only z component of magnetic field at the tip position, $H_x^{\text{inc}} = 0$ and $H_y^{\text{inc}} = 0$. Also, since the polarizability of the image dipole is proportional to the tip dipole $\alpha_{\text{tip}}^{\text{m}} \propto \alpha_{\text{img}}^{\text{m}}$, from Eq. (S18) the time-averaged optical force on the tip is related to the incident magnetic field at the tip position as $F_z \propto \left|\alpha_{\text{tip}}^{\text{m}} H_z^{\text{inc}}\right|^2$ which is Eq. (4) of the manuscript. Note that we have suppressed superscript "dipole" and subscript "m" in the notation of this section for simplicity.

Approximation condition of Eq. (4) with axis displacement for the real scenario with a truncated Si cone

We analyze the approximation condition of Eq. (4) in the main paper, i.e., the proportionality between the *magnetic force* and the *longitudinal magnetic field intensity*, even when the probe is slightly misaligned with respect to the axis of the incident APB. For this purpose, we define the *normalized longitudinal magnetic* field intensity to the maximum magnetic force as

$$\left|H_z^{\text{inc}}\right|^2 \frac{\text{Max}\left(F_{m,z}\right)}{\text{Max}\left(\left|H_z^{\text{inc}}\right|^2\right)}.$$

We perform the simulation of the realistic scenario as indicated in the main paper: the on-state truncated cone probe above the glass substrate is scanning in the transverse direction (*x*) around the axis of the incident APB, at 670 nm wavelength. The APB has a beam waist parameter of $w_0 = 0.7\lambda$ with incident beam power of 150 μW. We compare the normalized longitudinal magnetic field intensity and the longitudinal magnetic force with respect to the displacement *x* between the axis of the incident APB and the on-state probe, shown in Fig. S6. The light blue curve is the force due to induced magnetic dipole in the on-state truncated Si disk calculated by Eq. (5) whereas the dark blue curve is the longitudinal incident magnetic field intensity normalized to maximum force due to induced magnetic dipole.

We highlight the yellow region where the normalized longitudinal magnetic field and the magnetic force have an overlap accuracy (ratio) higher than 90%: the spot has a remarkable diameter close to 400 nm. Considering the wavelength for on-state magnetic excitation as 670 nm, the highlighted region shows a rather large area near APB axis where the proportionality between the longitudinal magnetic force and the incident magnetic field intensity holds.

The overlap accuracy indicated by the yellow highlight is related to the accuracy of the dipolar approximation upon a slight displacement between the axes of the incident beam and the probe. With a broken azimuthal symmetry, other multipoles rather than the desired magnetic dipole (i.e. electric dipole, electric/magnetic quadrupole etc) will be excited as shown in Fig S2, which affect

the overlap accuracy. Further improvement of the overlap accuracy requires the suppression of the excitation of additional multipoles. Such conditions can be achieved by utilizing magnetic probes with better isotropy, a smaller size and a stronger magnetic dipolar response, through improved design and fabrication techniques.

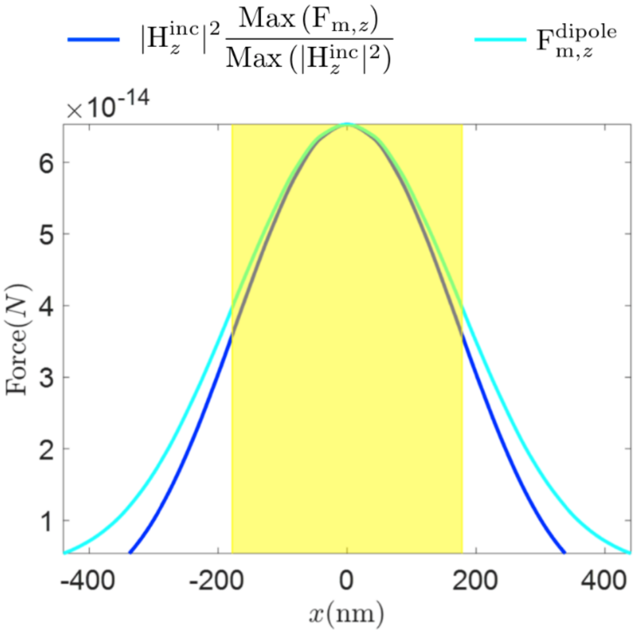

Fig. S5. Comparison of the normalized longitudinal magnetic incident field intensity and the longitudinal magnetic force. The force is exerted on the magnetic nanoprobe (on-state Si truncated cone) 5nm over the dielectric substrate versus displacement *x* with respect to the axis of the incident APB. The yellow highlight indicates the region where the ratio between the normalized longitudinal magnetic field intensity and the magnetic force is greater than 90%. The magnetic dipole force $F_{m,z}^{dipole}$ is calculated using Eq. (3).

Comparison between gold-coated probe and the Si probe

As described in the main manuscript, both the gold-coated sharp probe and blunt Si tip can serve as the electric probe to measure the electric force by the PiFM system. This is because the permittivity of both gold and Si allows electric dipole excitation by the light source in the probe, which gives rise to a nonzero dipole-mediated electric force.

However, there are important differences as well. Gold has a negative real permittivity and a small but non-negligible imaginary permittivity (loss) in the optical region. While Si also has low loss, relative to gold it has a smaller and positive permittivity. Consequently, the electric dipolar polarizability of gold tips is expected to be much stronger than for Si, producing stronger PiFM signals and thus superior SNR in the force detection measurement compared to a Si tip. The excellent polarizability of a gold probe can also enable the generation of detectable PiFM signals from small diameter tips (<10nm or even 1nm). On the other hand, a sharp Si probe may not have enough effective polarizability to induce a measurable electric force. Therefore, to enable efficient electric force detection, the blunt apex is necessary for the Si probe to provide a strong enough

electric dipole that is also close enough to the sample/substrate surface. This will naturally decrease the achievable resolution by the blunt Si probe. We provide a comparison between a PiFM measurement performed with a sharp gold tip (diameter<10nm) and a blunt Si probe (diameter~200nm) for the case of a tightly focused APB, which shows clear differences in signal strength and SNR in Fig. S7.

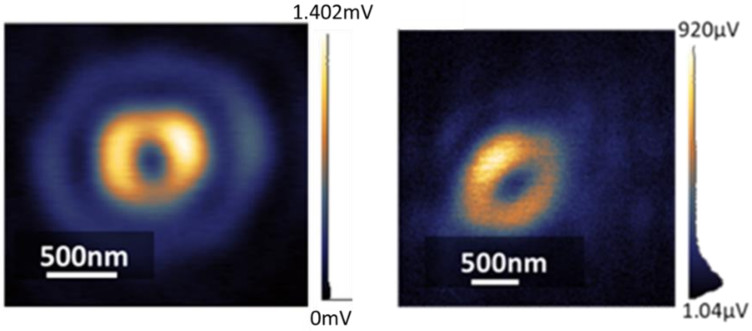

Fig. S6. The force maps of tightly focused APB. They are detected by the Left) sharp gold probe and Right) blunt Si probe, respectively. The force map detected by the sharp gold probe is from our previously published work, reprinted with permission from ref. (24). Copyright 2018 American Chemical Society.

REFERENCES AND NOTES

1. J. B. Pendry, Negative refraction makes a perfect lens. *Phys. Rev. Lett.* **85**, 3966–3969 (2000).

2. J. B. Pendry, A. Aubry, D. R. Smith, S. A. Maier, Transformation optics and subwavelength control of light. *Science* **337**, 549–552 (2012).

3. D. Nowak, W. Morrison, H. K. Wickramasinghe, J. Jahng, E. Potma, L. Wan, R. Ruiz, T. R. Albrecht, K. Schmidt, J. Frommer, D. P. Sanders, S. Park, Nanoscale chemical imaging by photoinduced force microscopy. *Sci. Adv.* **2**, e1501571 (2016).

4. M. Kasperczyk, S. Person, D. Ananias, L. D. Carlos, L. Novotny, Excitation of magnetic dipole transitions at optical frequencies. *Phys. Rev. Lett.* **114**, 163903 (2015).

5. M. Neugebauer, J. S. Eismann, T. Bauer, P. Banzer, Magnetic and electric transverse spin density of spatially confined light. *Phys. Rev. X* **8**, 021042 (2018).

6. U. Manna, J.-H. Lee, T.-S. Deng, J. Parker, N. Shepherd, Y. Weizmann, N. F. Scherer, Selective induction of optical magnetism. *Nano Lett.* **17**, 7196–7206 (2017).

7. N. Bonod, S. Bidault, G. W. Burr, M. Mivelle, Evolutionary optimization of all-dielectric magnetic nanoantennas. *Adv. Opt. Mater.* **7**, 1900121 (2019).

8. P. Banzer, U. Peschel, S. Quabis, G. Leuchs, On the experimental investigation of the electric and magnetic response of a single nano-structure. *Opt. Express* **18**, 10905–10923 (2010).

9. E. Betzig, R. J. Chichester, Single molecules observed by nearfield scanning optical microscopy. *Science* **262**, 1422–1425 (1993).

10. C. M. Dodson, R. Zia, Magnetic dipole and electric quadrupole transitions in the trivalent lanthanide series: Calculated emission rates and oscillator strengths. *Phys. Rev. B* **86**, 125102 (2012).

11. C. D. Stanciu, F. Hansteen, A. V. Kimel, A. Kirilyuk, A. Tsukamoto, A. Itoh, T. Rasing, All-optical magnetic recording with circularly polarized light. *Phys. Rev. Lett.* **99**, 047601 (2007).

12. K. C. Neuman, A. Nagy, Single-molecule force spectroscopy: optical tweezers, magnetic tweezers and atomic force microscopy. *Nat. Methods* **5**, 491–505 (2008).

13. M. Nieto-Vesperinas, J. J. Sáenz, R. Gómez-Medina, L. Chantada, Optical forces on small magnetodielectric particles. *Opt. Express* **18**, 11428–11443 (2010).

14. V. M. Shalaev, W. Cai, U. K. Chettiar, H.-K. Yuan, A. K. Sarychev, V. P. Drachev, A. V. Kildishev, Negative index of refraction in optical metamaterials. *Opt. Lett.* **30**, 3356–3358 (2005).

15. G. Dolling, C. Enkrich, M. Wegener, J. F. Zhou, C. M. Soukoulis, S. Linden, Cut-wire pairs and plate pairs as magnetic atoms for optical metamaterials. *Opt. Lett.* **30**, 3198–3200 (2005).

16. E. Devaux, A. Dereux, E. Bourillot, J. Weeber, Y. Lacroute, J. Goudonnet, C. Girard, Local detection of the optical magnetic field in the near zone of dielectric samples. *Phys. Rev. B* **62,** 10504–10514 (2000).

17. M. Burresi, D. Van Oosten, T. Kampfrath, H. Schoenmaker, R. Heideman, A. Leinse, L. Kuipers, Probing the magnetic field of light at optical frequencies. *Science* **326**, 550–553 (2009).

18. H. Giessen, R. Vogelgesang, Glimpsing the weak magnetic field of light. *Science* **326**, 529–530 (2009).

19. B. Le Feber, N. Rotenberg, D. M. Beggs, L. Kuipers, Simultaneous measurement of nanoscale electric and magnetic optical fields. *Nat. Photonics* **8**, 43–46 (2014).

20. M. Poblet, Y. Li, E. Cortés, S. A. Maier, G. Grinblat, A. V. Bragas, Direct detection of optical forces of magnetic nature in dielectric nanoantennas. *Nano Lett.* **20**, 7627–7634 (2020).

21. C. Guclu, V. A. Tamma, H. K. Wickramasinghe, F. Capolino, Photoinduced magnetic force between nanostructures. *Phys. Rev. B.* **92**, 235111 (2015).

22. C. Guclu, M. Veysi, F. Capolino, Photoinduced magnetic nanoprobe excited by an azimuthally polarized vector beam. *ACS Photonics* **3**, 2049–2058 (2016).

23. J. Zeng, F. Huang, C. Guclu, M. Veysi, M. Albooyeh, H. K. Wickramasinghe, F. Capolino, Sharply focused azimuthally polarized beams with magnetic dominance: Nearfield characterization at nanoscale by photoinduced force microscopy. *ACS Photonics* **5**, 390–397 (2017).

24. J. Zeng, M. Darvishzadeh-Varcheie, M. Albooyeh, M. Rajaei, M. Kamandi, M. Veysi, E. O. Potma, F. Capolino, H. K. Wickramasinghe, Exclusive magnetic excitation enabled by structured light illumination in a nanoscale mie resonator. *ACS Nano* **12**, 12159–12168 (2018).

25. I. Rajapaksa, K. Uenal, H. K. Wickramasinghe, Image force microscopy of molecular resonance: A microscope principle. *Appl. Phys. Lett.* **97**, 073121 (2010).

26. J. Jahng, J. Brocious, D. A. Fishman, F. Huang, X. Li, V. A. Tamma, H. K. Wickramasinghe, E. O. Potma, Gradient and scattering forces in photoinduced force microscopy. *Phys. Rev. B.* **90**, 155417 (2014).

27. M. A. Almajhadi, S. M. A. Uddin, H. K. Wickramasinghe, in *2020 Conference on Lasers and Electro-Optics (CLEO)* (IEEE, 2020), pp. 1–2.

28. I. Staude, A. E. Miroshnichenko, M. Decker, N. T. Fofang, S. Liu, E. Gonzales, J. Dominguez, T. S. Luk, D. N. Neshev, I. Brener, Tailoring directional scattering through magnetic and electric resonances in subwavelength silicon nanodisks. *ACS Nano* **7**, 7824–7832 (2013).

29. A. I. Kuznetsov, A. E. Miroshnichenko, M. L. Brongersma, Y. S. Kivshar, B. Luk'yanchuk, Optically resonant dielectric nanostructures. *Science* **354**, aag2472 (2016).

30. M. Veysi, C. Guclu, F. Capolino, Vortex beams with strong longitudinally polarized magnetic field and their generation by using metasurfaces. *J. Opt. Soc. Am. B* **32**, 345–354 (2015).

31. M. Veysi, C. Guclu, F. Capolino, Focused azimuthally polarized vector beam and spatial magnetic resolution below the diffraction limit. *J. Opt. Soc. Am. B* **33**, 2265–2277 (2016).

32. A. D. Yaghjian, in *IEEE Antennas and Propagation Society International Symposium. 1999 Digest. Held in conjunction with: USNC/URSI National Radio Science Meeting (Cat. No. 99CH37010)* (IEEE, 1999), vol. 4, pp. 2868–2871.

33. M. N. O. Sadiku, *Computational Electromagnetics with MATLAB, Fourth Edition* (CRC Press LLC, 2018).

34. COMSOL Multiphysics® Modeling Software; www.comsol.com/.

35. M. Rajaei, M. A. Almajhadi, J. Zeng, H. K. Wickramasinghe, Nearfield nanoprobing using Si tip-Au nanoparticle photoinduced force microscopy with 120:1 signal-to-noise ratio, sub-6-nm resolution. *Opt. Express* **26**, 26365–26376 (2018).

36. C. Debus, M. A. Lieb, A. Drechsler, A. J. Meixner, Probing highly confined optical fields in the focal region of a high NA parabolic mirror with subwavelength spatial resolution. *J. Microsc.* **210**, 203–208 (2003).

37. G. M. Lerman, A. Yanai, U. Levy, Demonstration of nanofocusing by the use of plasmonic lens illuminated with radially polarized light. *Nano Lett.* **9**, 2139–2143 (2009).

38. F. Zenhausern, Y. Martin, H. K. Wickramasinghe, Scanning interferometric apertureless microscopy: Optical imaging at 10 angstrom resolution. *Science* **269**, 1083–1085 (1995).

39. M. Sanz-Paz, C. Ernandes, J. U. Esparza, G. W. Burr, N. F. van Hulst, A. Maitre, L. Aigouy, T. Gacoin, N. Bonod, M. F. Garcia-Parajo, Enhancing magnetic light emission with all-dielectric optical nanoantennas. *Nano Lett.* **18**, 3481–3487 (2018).

40. D. E. Aspnes, A. A. Studna, Dielectric functions and optical parameters of Si, Ge, Gap, GaAs, GaSb, InP, InAs, and InSb from 1.5 to 6.0 eV. *Phys. Rev. B* **27**, 985 (1983).

41. J. Jahng, E. O. Potma, E. S. Lee, Tip-enhanced thermal expansion force for nanoscale chemical imaging and spectroscopy in photoinduced force microscopy. *Anal. Chem.* **90**, 11054–11061 (2018).

42. J. D. Jackson, *Classical Electrodynamics Third Edition* (Wiley, ed. 3, 1998).

43. J. Chen, J. Ng, Z. Lin, C. T. Chan, Optical pulling force. *Nat. Photonics* **5**, 531–534 (2011).

44. R. E. Raab, O. L. de Lange, *Multipole Theory in Electromagnetism: Classical, Quantum, and Symmetry Aspects, with Applications* (Oxford Univ. Press, 2004).

45. E. E. Radescu, G. Vaman, Exact calculation of the angular momentum loss, recoil force, and radiation intensity for an arbitrary source in terms of electric, magnetic, and toroid multipoles. *Phys. Rev. E* **65**, 046609 (2002).

46. R. Alaee, C. Rockstuhl, I. Fernandez-Corbaton, An electromagnetic multipole expansion beyond the long-wavelength approximation. *Opt. Commun.* **407**, 17–21 (2018).

47. Z. Y. Wang, R. J. Zhang, S. Y. Wang, M. Lu, X. Chen, Y. X. Zheng, L. Y. Chen, Z. Ye, C. Z. Wang, K. M. Ho, Broadband optical absorption by tunable Mie resonances in silicon nanocone arrays. *Sci. Rep.* **5**, 1–6 (2015).

48. E. Kim, Y. Cho, K. Park, J. Choi, S. Lim, Y. Cho, Y. Nam, J. Lee, D. Kim, Mie resonance-mediated antireflection effects of Si nanocone arrays fabricated pm 8-in. wafers using a nanoimprint technique. *Nanoscale Res. Lett.* **10**, 164 (2015).
